# Apropos: *Plasmodium knowlesi* malaria an emerging public health problem in Hulu Selangor, Selangor, Malaysia (2009–2013): epidemiologic and entomologic analysis

**DOI:** 10.1186/s13071-015-0694-8

**Published:** 2015-02-05

**Authors:** Sheldon Waugh

**Affiliations:** Department of Epidemiology, College of Public Health and Health Professions, University of Florida, Gainesville, FL 32610 USA

**Keywords:** GIS, Spatial, Kernel, Analysis, Malaria, Methodology

## Abstract

The use of detailed methodologies and legitimate settings justifications in spatial analysis is imperative to locating areas of significance. Studies missing this action may enact interventions in improper areas.

## Apropos: *Plasmodium knowlesi* malaria an emerging public health problem in Hulu Selangor, Selangor, Malaysia (2009–2013): epidemiologic and entomologic analysis

The study authored by Vythilingam *et al*. brings new insight; producing new information to the increased incidence of *Plasmodium knowlesi* and *Plasmodium malariae* in NE Sengalor [1]. However, there are some flaws present within mosquito sampling and methodology for spatial analysis that affect the possible results and conclusions reached by the authors.

A look into the mosquito and human case collection methods, shows a discrepancy between the time in the collection of mosquitoes (2012-2013) and the human case reviews (2009-2013). This may not allow for the authors to make assumptions and conclusions about the possible vectors outside of the mosquito collection period of 2012 to 2013. Greater care must be taken by the authors to make conclusions that account for the difference in collection periods.

Next, topics of concern lie within the methodology of how the spatial analysis was conducted for hotspot detection of *P. knowlesi* and *P. malariae* human cases. Overall, the study’s spatial analysis did not apply a through enough methodology, in locating human case hotspots of malaria, which may shift the spatial distribution towards a more accurate depiction of disease distribution. The created hotspot map demonstrated the use of a kernel density tool to locate hotspots, within the study area; however, their analysis failed to include a more detailed explanation in the context of settings, within this tool.

**Figure 1:**
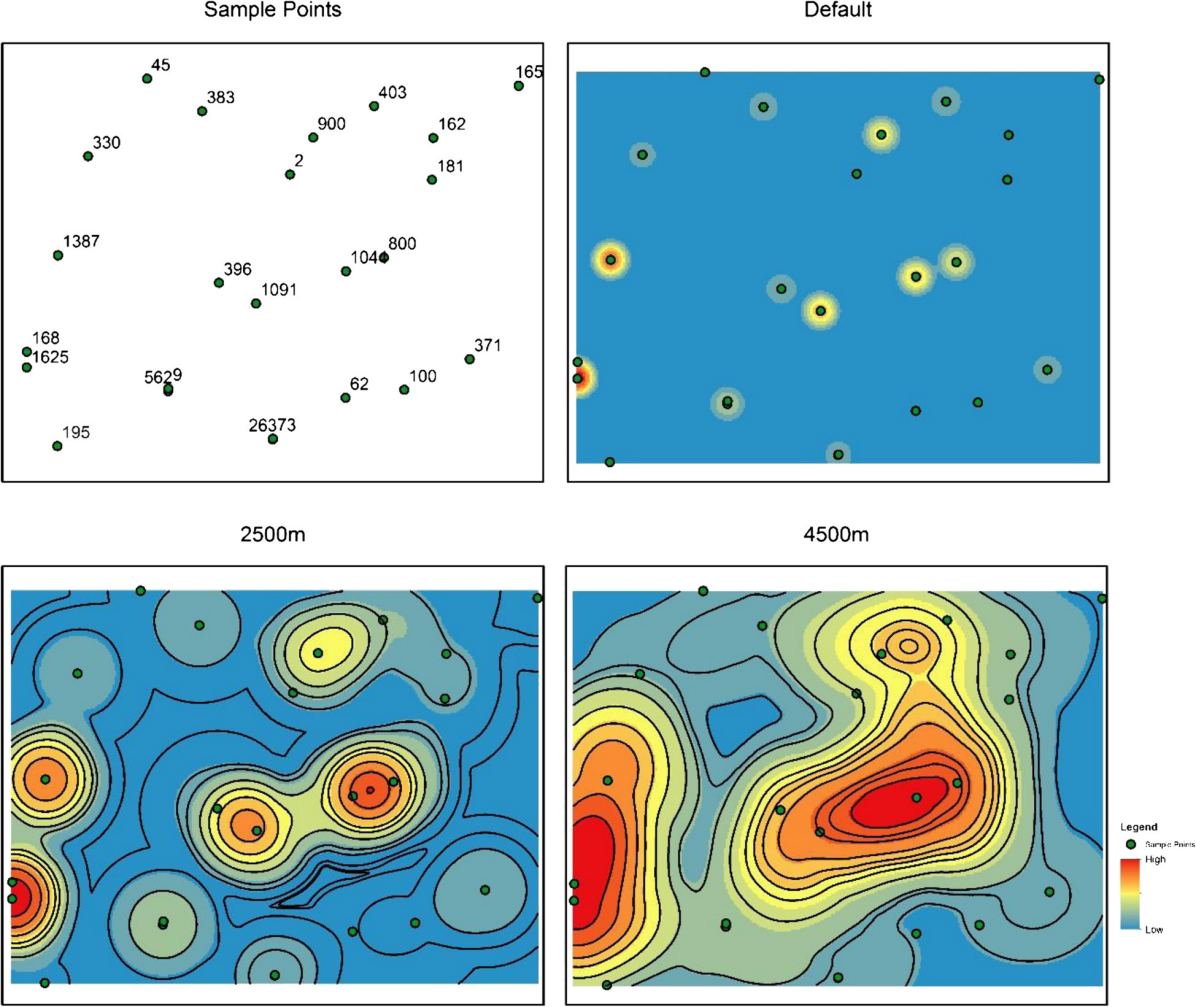
**Spatial representation of varying search radii for ArcGIS 10.2.2 kernel density tool.**

The Kernel Density tool for ArcGIS 9.3 uses settings and default specifications that are determined using the spatial extent limitations of the data given (the default search radius was calculated by taking the smaller of the width or height of the extent of the input, divided by 30). The use of the default search radius (or other search radii) will not affect the numerical calculation of the density function, but the spatial visualization of the tool will change; creating a more generalized and smoothed surface (Figure 1). This could lead to significant bias that leads to possible hotspots, within Hulu Selangor, not being logged or correctly identified.

Spatial tools, such as kernel density, have sensitive settings that require explanation and reasoning behind the specified use. The authors should have combined the use of the Kernel Density tool along with other statistical or mathematical analysis tools, such as that in ArcGIS 10.2.2, that uses a search radius default equation that takes the mean center and standard deviation of the data points into consideration. The use of Optimized Hot-Spot Analysis is also entirely possible with the amount of total data points from 2009-2013. Optimized Hot-Spot Analysis aggregates incident points into grid-like polygons and conducts a Getis-Ord Gi* Hotspot Analysis on those polygons to determine visual hotspots of data points [2].

Another point to consider is the use of an organized methodology for spatial analysis. Dom et al. uses spatial analysis with an organized spatial methodology to detect hotspots of dengue within Subang Jaya, Malaysia [3]. They provided a detailed methodology for the settings of their kernel analysis and other spatial tools, which brings clarity and shows a high regard for these robust tools. In addition to Dom et al., there are multiple examples of kernel density using multiples justifications and methodologies that range from the use of reasonable causal information [4] to testing of multiple search radii using statistical methods [5].

The possibility of bias due to poor applications of spatial analysis is widely present within the sub-discipline of spatial epidemiology. The improper display of spatial data may lead to improper allotment of funds and resources to areas that may have been incorrectly identified as hotspots. Further spatial-related research must take greater care and deference when using tools and estimators to avoid these problems in the future.
